# Osteoporosis in children and adolescents: when to suspect and how to diagnose it

**DOI:** 10.1007/s00431-022-04455-2

**Published:** 2022-04-06

**Authors:** Silvia Ciancia, Rick R. van Rijn, Wolfgang Högler, Natasha M. Appelman-Dijkstra, Annemieke M. Boot, Theo C. J. Sas, Judith S. Renes

**Affiliations:** 1 Department of Pediatrics, Subdivision of Endocrinology, Erasmus University Medical Center, Sophia Children’s Hospital, Rotterdam, The Netherlands; 2 Department of Radiology and Nuclear Medicine, Amsterdam UMC, University of Amsterdam, Amsterdam, The Netherlands; 3 Department of Paediatrics and Adolescent Medicine, Johannes Kepler University Linz, Linz, Austria; 4 Department of Internal Medicine, Subdivision of Endocrinology, Center for Bone Quality, Leiden University Medical Center, Leiden, The Netherlands; 5 Department of Pediatrics, Subdivision of Endocrinology, University Medical Center Groningen, Beatrix Children’s Hospital, University of Groningen, Groningen, The Netherlands; 6 Diabeter, Center for Pediatric and Adult Diabetes Care and Research, Rotterdam, The Netherlands

**Keywords:** Osteoporosis, Primary osteoporosis, Secondary osteoporosis, Pediatrics, Bone health, DXA

## Abstract

Early recognition of osteoporosis in children and adolescents is important in order to establish an appropriate diagnosis of the underlying condition and to initiate treatment if necessary. In this review, we present the diagnostic work-up, and its pitfalls, of pediatric patients suspected of osteoporosis including a careful collection of the medical and personal history, a complete physical examination, biochemical data, molecular genetics, and imaging techniques. The most recent and relevant literature has been reviewed to offer a broad overview on the topic. Genetic and acquired pediatric bone disorders are relatively common and cause substantial morbidity. In recent years, there has been significant progress in the understanding of the genetic and molecular mechanistic basis of bone fragility and in the identification of acquired causes of osteoporosis in children. Specifically, drugs that can negatively impact bone health (e.g. steroids) and immobilization related to acute and chronic diseases (e.g. Duchenne muscular dystrophy) represent major risk factors for the development of secondary osteoporosis and therefore an indication to screen for bone mineral density and vertebral fractures. Long-term studies in children chronically treated with steroids have resulted in the development of systematic approaches to diagnose and manage pediatric osteoporosis.

*Conclusions*: Osteoporosis in children requires consultation with and/or referral to a pediatric bone specialist. This is particularly relevant since children possess the unique ability for spontaneous and medication-assisted recovery, including reshaping of vertebral fractures. As such, pediatricians have an opportunity to improve bone mass accrual and musculoskeletal health in osteoporotic children.

**Table:** 

**What is Known:** *• Both genetic and acquired pediatric disorders can compromise bone health and predispose to fractures early in life.* *• The identification of children at risk of osteoporosis is essential to make a timely diagnosis and start the treatment, if necessary.*	
**What is New:** *• Pediatricians have an opportunity to improve bone mass accrual and musculoskeletal health in osteoporotic children and children at risk of osteoporosis.* *• We offer an extensive but concise overview about the risk factors for osteoporosis and the diagnostic work-up (and its pitfalls) of pediatric patients suspected of osteoporosis.*

**What is Known:**

*• Both genetic and acquired pediatric disorders can compromise bone health and predispose to fractures early in life.*

*• The identification of children at risk of osteoporosis is essential to make a timely diagnosis and start the treatment, if necessary.*

**What is New:**

*• Pediatricians have an opportunity to improve bone mass accrual and musculoskeletal health in osteoporotic children and children at risk of osteoporosis.*

*• We offer an extensive but concise overview about the risk factors for osteoporosis and the diagnostic work-up (and its pitfalls) of pediatric patients suspected of osteoporosis.*

## Introduction

Over the last few decades, osteoporosis in children has been increasingly recognized. Both genetic and acquired pediatric bone disorders can compromise bone strength leading to fractures during childhood. If left untreated, these conditions lead to reduced bone mass, deformities, and impact quality of life, with potential long-term consequences [1, 2]. Awareness among pediatricians is therefore important to identify patients with, or at risk of developing, osteoporosis.

Before the age of 18 years, approximately 95% of the skeletal size and bone and muscle mass is acquired [3]. Therefore, childhood is a very important time to build a strong musculoskeletal system. Factors influencing bone structure and quality are genetic background, organ function, chronic systemic illnesses, medications, and muscular disorders as well as metabolic disorders. Primary osteoporosis usually occurs due to an underlying genetic defect. The most common condition is osteogenesis imperfecta (OI) [4, 5] and more than 24 genes have been identified which cause OI [6]. Secondary, or acquired, osteoporosis develops in children and adults with chronic systemic illnesses due to effects of the disease itself or its treatment. Children and adolescents with osteoporosis can present with a history of recurrent fractures, deformities, or back pain. The accidental finding of vertebral fractures (VFs) on lateral spine radiographs can also lead to the diagnosis of osteoporosis [7, 8].

In both primary and secondary osteoporosis, hidden vertebral fractures delay the diagnosis of osteoporosis. Early identification of VFs through lateral spine imaging should lead to referral to a specialist, because early treatment has the potential to prevent future fractures. In this review, we will discuss normal bone physiology, the definition of osteoporosis and will particularly focus on the diagnostic work-up, and its pitfalls, of children suspected of osteoporosis using clinical signs, biochemistry, molecular genetic testing, and imaging techniques.

## Normal bone physiology

Bone is a dynamic tissue, and the growth in length and width (modeling) and the remodeling of bone are complex processes. Here we will discuss the key features.

Bone is composed of minerals (50–70%, mostly hydroxyapatite), organic matrix (20–40%, mostly collagen), water (5–10%), and lipids (< 3%) [9]. The mineral content lends strength and rigidity to the bone, while the organic matrix is responsible for its elasticity and toughness [10, 11]. Most of the skeleton consists of cortical bone (~ 80%), while the inner skeletal compartment is composed of a honeycomb-like structure known as trabecular bone. Although trabecular bone represents only 20% of total bone mass, its surface area is far greater than that of cortical bone and its turnover is more dynamic. Consequently, bone loss in regions that are mainly composed of the more metabolically active trabecular bone (e.g. vertebrae, hip) are more susceptible for true osteoporotic fractures [12].

Bone tissue contains several cell types, namely, osteoclasts (break down bone matrix), osteoblasts (promote formation of new bone tissue), osteocytes (orchestrate the activity of osteoclasts and osteoblasts in response to mechanical strain, and also build bone), osteomorphs (involved in the regulation of bone resorption), and bone lining cells [13–16]. Bone modelling summarizes all the processes involved in growth and shaping of new bone, including the bone formation needed for bone elongation and widening (growth), metaphyseal inwaisting (the shaping of the end of long bones), and modelling drift of pelvic bone. Bone formation by osteoblasts or osteocytes includes the secretion of osteoid, mostly consisting of type I collagen, and its mineralization to form mature bone matrix. Bone remodeling involves old-by-new replacement in three consecutive phases with osteoclast-mediated resorption of existing bone and the consequent release of calcium and phosphate, the reversal phase in which osteoblast cells appear on the bone surface, and lastly, the osteoblast-mediated synthesis of osteoid that will undergo mineralization to form mature bone matrix [14, 16].

The molecular pathways that regulate bone formation, resorption, and remodeling are complex and their discussion is beyond the scope of this review. We will briefly focus on the RANK (Receptor Activator of Nuclear Factor Kappa B)–RANKL (RANK Ligand) system, WNT-signaling (Wingless iNTegration site family), and TGF-β (Transforming Growth Factor-β) signaling pathway.

One of the principal regulatory pathways is the RANK–RANKL–OPG (osteoprotegerin) system. RANK is expressed on the surface of osteoclast precursors and RANKL is expressed by osteoblasts and osteocytes. When RANKL binds with RANK, cell differentiation of osteoclast precursors is activated and consequently osteoclast-mediated bone resorption [17]. Osteoblasts also express OPG, a decoy receptor that binds to RANKL. By preventing the interaction between RANK and RANKL, RANK activation is inhibited and bone resorption is prevented [18]. Systemic regulators involved in this pathway include, amongst others, parathyroid hormone (PTH), active vitamin D, glucocorticoids, growth hormone, and sex hormones. Also, cytokines such as interleukin (IL)-1, IL-6, and tumor necrosis factor can activate this bone resorption pathway.

The WNT signaling pathway promotes osteogenesis and increases bone mass by suppressing apoptosis in osteoblast precursor cells and facilitating osteoblast differentiation. The WNT pathway is activated by ligands, such as Wnt1 and Wnt3a, through their binding to the transmembrane Frizzled receptors and LRP (low-density lipoprotein receptor-related protein)-5 and LRP-6 complexes. As OPG opposes RANK, sclerostin (secreted by osteocytes) inhibits the WNT signaling pathway through its binding to LRP-5 and LRP-6 [19].

Osteogenesis is enhanced by the TGF-β signaling pathway that promotes the recruitment, proliferation and differentiations of progenitor cells into osteoblasts. TGF-β is mainly secreted by the extracellular matrix and osteoclasts can increase its secretion to balance bone resorption. Also, the TGF-β pathway interacts with the WNT signaling through the inhibition of sclerostin secretion and the upregulations of several WNT ligands [20, 21].

## Definition of osteoporosis

According to the International Society for Clinical Densitometry (ISCD), pediatric osteoporosis is currently defined by (1) the combination of a bone mineral density (BMD) *Z*-score ≤ −2 and a clinically significant fracture history defined as the presence of either two or more long bone fractures before the age of 10 years or three or more long bone fractures at any age up to 19 years; or (2) one or more vertebral compression fractures occurring without high energy trauma or local disease irrespective of the BMD *Z*-score [22, 23].

As childhood fractures are very common [24–26], this definition aims to distinguish children with an underlying condition from those who experience fractures as a result of typical childhood behavior or non-accidental trauma. There are, however, several challenges in using this definition. For example, the inclusion of a BMD *Z*-score cut-off of ≤ –2 in defining osteoporosis. Depending on the reference data used to calculate the BMD *Z*-score, this score can differ by as much as 2 SD [27–29]. Another challenge is the risk of underdiagnosing conditions predisposing to osteoporosis, e.g. whilst waiting for the second or third fracture in children with low BMD or because the BMD *Z*-score is above −2 despite recurrent fractures. Therefore, in line with current recommendations, diagnosing osteoporosis should not be based on BMD alone but take into account the clinical context, specifically the severity and prognosis of the underlying disease or treatment [30].

## Primary osteoporosis

Primary osteoporosis refers to conditions of heritable bone fragility caused by intrinsic skeletal defects with abnormal composition of bone tissue. Causative genes affect different pathways such as collagen type I synthesis, bone mineralization, osteoblasts, or osteocyte dysfunction [31, 32]. Children with primary osteoporosis comprise a heterogeneous group with a broad spectrum of skeletal and extraskeletal characteristics, ranging from mild to lethal forms. These conditions result in severe bone disease and low bone mass accrual. Timely recognition is therefore important to initiate treatment and specialist care [33–35].

OI is the most common form of primary osteoporosis. The main clinical features are recurrent fractures, skeletal deformities, short stature, blue sclera, dentinogenesis imperfecta, hearing loss, and ligamentous laxity; however, these can vary among patients depending on the type of OI [36]. Inheritance of the most frequent types of OI is autosomal dominant (type 1–5; 85–90% are caused by *COL1A1*, *COL1A2*, or *IFITM5* mutations), while rarer forms show autosomal recessive or X-linked inheritance [4, 36–38]. More rare primary osteoporotic conditions are described in Table 1 [33, 39–45].

**Table 1:** Primary osteoporosis: pathways involved, conditions, genes involved and inheritance. Adapted from El-Gazzar et al. [13]. OI: Osteogenesis Imperfecta. Clinical types of OI: I mild (OI 1,14,16), II perinatal lethal (OI 2,7,8,9), III severe (OI 3,6,7,8,9,10,11,13,14,15,16,17,18,19,20), IV moderate (OI 4,5,7,11,12,14,15,17,19)

**Primary osteoporosis**			
**Bone pathways**	**Conditions**	**Genes**	**Inheritance**
**Osteogenesis imperfecta and other forms of primary osteoporosis**			
** Collagen synthesis**	OI 1,2,3,4	*COL1A1-COL1A2*	AD
** Collagen folding and cross-linking**	OI 10	*SERPINH1*	AR
	OI 11, Bruck Syndrome Type 1	*FKBP10*	AR
	Bruck Syndrome Type 2 (BS2)	*PLOD2*	AR
** Collagen modification**	OI 7	*CRTAP*	AR
	OI 8	*LEPRE1 (P3H1)*	AR
	OI 9	*PPIB*	AR
** Procollagen/collagen processing**	OI 13	*BMP1*	AR
	OI 17	*SPARC*	AR
** Mineralization**	OI 5	*IFITM5*	AD
	OI 6	*SERPINF 1*	AR
	Calvarial doughnut lesions with bone fragility without (CDL) or with spondylometaphyseal dysplasia (CDLSMD)	*SGMS2*	AD
** Osteoblast differentiation and maturation**	OI 12	*SP7*	AR
** ER calcium flux**	OI 14	*TMEM38B*	AR
** ER UPR response, ER-Golgi trafficking**	OI 16	*CREB3L1*	AR or AD
	OI clinical type III	*KDELR2*	AR
** ER COPII transport of procollagen**	OI clinical type III (overlap with Cole-Carpenter Syndrome 2)	*SEC24D*	AR
** Golgi-regulated intramembrane proteolysis**	OI 19	*MBTPS2*	XL
** WNT signaling**	OI 15	*WNT1*	AR
	Primary osteoporosis	*WNT1*	AD
	OI 20	*MESD*	AR
	Osteoporosis pseudoglioma syndrome	*LRP5*	AR
	Primary osteoporosis	*LRP5*	AD
** BMP signaling**	OI 18 overlap with Stuve-Wiedemann syndrome	*TENT5A (FAM46A)*	AR
** TGF-ß pathway**	Loeys-Dietz syndrome	*SMAD3*	AD
** MAPK pathway**	OI clinical type III	*CCDC134*	AR
** Formation of F-actin bundles**	Primary osteoporosis	*PLS3*	XL
** Catalyzes rearrangement of disulfide bonds**	Cole-Carpenter syndrome 1	*P4HB*	AD
** Proteoglycan biosynthesis**	Spondylo-ocular dysplasia	*XYLT2*	AR
** Unclear**	Cutis laxa (ARCL2B)	*PYCR1*	AR
	Geroderma osteodysplasticum	*GORAB*	AR
	Gnathodiaphyseal dysplasia	*ANO5*	AD
	Singleton-Mertin dysplasia type 1	*IFIH1*	AD
	Singleton-Mertin dysplasia type 2	*DDX58*	AD
	Spinal muscular atrophy with congenital bone fractures-1 (SMABF1)	*TRIP4*	AR
	Spinal muscular atrophy with congenital bone fractures-2 (SMABF2)	*ASCC1*	AR
**Osteolytic forms**			
** RANK overactivation**	Familial expansile osteolysis (FEO) Juvenile Paget’s Disease (PDB2)	*TNFRSF11A*	AD
** OPG deficiency with** ** Increased RANKL-mediated osteoclastogenesis**	Juvenile Paget’s Disease (PDB5)	*TNFRSF11B*	AR
** Regulate cell fate; osteoblast and osteoclast function**	Hajdu-Cheney Syndrome	NOTCH2	AD
** Unknown**	Multicentric osteolysis, nodulosis, and arthropathy (MANO)	MMP2-MMP14	AR

*AD* autosomal dominant, *AR* autosomal recessive, *XL* X-linked, *BMP* bone morphogenetic protein, *ER* endoplasmic reticulum, *MAPK* mitogen-activated protein kinase, *OPG* osteoprotegerin, *RANK* receptor activator of NF-KappaB, *TGF* transforming growth factor, *UPR* unfolded protein response, *WNT* wingless-related integration site

In contrast, Idiopathic Juvenile Osteoporosis (IJO) is a condition with unknown pathophysiology. IJO is characterized by pain in the back, hips and/or lower limbs and difficulty walking, as well as vertebral compression fractures and long bone fractures. The onset of symptoms is insidious and usually starts before puberty. Interestingly, during puberty the symptoms may improve [46, 47]. In contrast to most genetic causes of osteoporosis, there is no positive family history, no extraskeletal manifestations and no growth impairment. With further molecular genetic advancements, the diagnosis of IJO is expected to diminish. Already, heterozygous mutations of *LRP5* have been described in some cases [48]. To date, the diagnosis of IJO remains a clinical one and based on exclusions of other causes of osteoporosis [46, 47].

## Secondary osteoporosis

Secondary osteoporosis occurs as a result of systemic underlying conditions or medications. The most common causes include inflammatory disorders, hematological and oncological disorders, renal disease, immobility or muscle impairment and medications such as corticosteroids (Table 2). At any age, malnutrition, immobilization, and lack of physical activity represent additional risk factors for osteoporosis development [7, 49–57].

**Table 2:** Main conditions associated to secondary osteoporosis

**Secondary osteoporosis**
**Endocrine disorders**
Hypercortisolism
Hyperthyroidism
Hypogonadism (e.g. hypopituitarism, Turner syndrome, Klinefelter syndrome)
**Gastro-intestinal disorders**
Inflammatory bowel disease
Malabsorption syndromes (e.g. cholestatic liver failure, celiac disease, cystic fibrosis)
Short bowel syndrome
**Cytokine-induced osteoporosis**
Leukemia
Juvenile idiopathic arthritis
Systemic lupus erythematosus
**Medications**
Anticonvulsants
Chemotherapy
Glucocorticoids
**Immobility-induced osteoporosis**
Duchenne muscular dystrophy
Cerebral palsy

Depending on the underlying cause, the pathophysiology of osteoporosis differs. For example, 16% of children with acute lymphoblastic leukemia already have VFs at diagnosis, likely caused by the release of cytokines from leukemic cells that stimulate osteoclast activity [8]. The risk of fractures is the highest in the first two years of diagnosis and the presence of VFs at diagnosis is highly predictive of future fractures. Up to 45% of children presenting with VFs at the diagnosis can be asymptomatic; therefore, lateral spine imaging is advised to detect asymptomatic VFs, predict the risk of future fractures, and prevent vertebral deformities and long-term morbidity [58–60].

Immobility is another frequent cause of osteoporosis. According to the mechanostat theory, bone strength is regulated by muscle force. During immobilization, lack of muscle tension results in reduced biomechanical bone loading, which is sensed by osteocytes and translated into biochemical signals that lead to thinner long bones and less trabecular bone formation [7, 13, 61]. Hence, children with for example cerebral palsy have reduced periosteal apposition in lower extremity bones, resulting in reduced cortical thickness. Consequently, fractures occur most commonly in the distal femur and tibia [62–64]. Also, children affected by Duchenne Muscular Dystrophy require a careful follow-up by a pediatric bone specialist because of immobility, long-term steroid treatment, and hypogonadism [63, 65].

The detrimental effect of glucocorticosteroids (GCs) on bone is caused by an initial phase of increased bone resorption followed by a phase of decreased bone formation [66–69]. In children, high cumulative doses of intravenous and/or oral corticosteroids and repeated pulse therapy have been associated with the development of osteoporosis [70]. There are no conclusive data available on the effect of low and medium doses of steroids; however, from data acquired in the adult population, the chronic administration of systemic corticosteroids at a medium to low dose is also suspected to impair growth and affect bone formation [71, 72]. Furthermore, long-term inhaled corticosteroid (ICS) therapy in children may negatively affect BMD. This was seen in children treated with high doses of ICS but not in children treated with low and medium doses [73–75]. However, fracture rate is not increased in children on ICS when adjusted for asthma severity [76].

With improving survival rates in many systemic conditions, complications such as osteoporosis are on the rise, and hence, monitoring of bone health should be part of the standardized follow-up [77]. In some subjects presenting with low impact fractures or back pain, however, the underlying disease is not yet known and they present with signs of impaired bone health.

## Clinical signs and laboratory work-up

In a child suspected of or with osteoporosis, a thorough medical history needs to be taken. History of fractures (number, localization, mechanism, and radiographic features) must be investigated. Back pain needs to be questioned because it might be a sign of vertebral fractures. Furthermore, a detailed history of comorbidities, physical activity, diet and medications, growth and puberty, and family history (e.g. fractures, hearing loss) needs to be taken. Physical examination should include anthropometry including head circumference, body proportions, assessment of teeth, sclera, joint laxity, scoliosis, limb deformities, widening of the wrists and ankles, spine tenderness, skin laxity, and pubertal status.

In every child referred for assessment of bone health-selected laboratory studies of bone mineralization should be performed including serum calcium, phosphate, magnesium, creatinine, alkaline phosphatase (ALP), gamma glutamyl transferase (GGT), 25-hydroxy vitamin D, PTH, and urinary creatinine, calcium, and phosphate. Whilst this biochemical assessment excludes bone hypomineralization disorders (all types of rickets/osteomalacia), there is no current blood test that can diagnose or exclude osteoporosis apart from molecular genetic testing. Where no secondary cause of osteoporosis can be found, targeted-, whole exome-, and RNA sequencing methods should be considered to search for a genetic cause [78].

In rare cases, fractures or bone pain may be the first presenting symptom of an underlying condition. Therefore, erythrocyte sedimentation rate, a full blood count with leucocyte differentiation, serum TSH, free T4, and celiac screening is also advised. If hypogonadism is suspected, the LH, FSH, testosterone (♂), or estradiol (♀) should be checked; if Cushing’s disease is suspected, 24-h urinary cortisol should be checked. This work-up should be tailored to the presenting symptoms and population-appropriate pediatric reference data used [7, 39].

## Diagnostic techniques to assess bone health

The main imaging techniques used to assess bone health in children are dual-energy X-ray absorptiometry (DXA) and conventional lateral spine radiographs. The other techniques described below are mainly reserved for research.

### Dual-energy X-ray absorptiometry

Dual-energy X-ray absorptiometry (DXA) is the most commonly used technique for assessing bone mass in children due to its worldwide availability, precision, reproducibility, and availability of normative data [23, 79]. In children, the preferred measurement sites are the lumbar spine (LS) and the total body less head (TBLH) [23, 80]. DXA-derived values for children are expressed as age-specific and sex-specific *Z*-scores. Normative pediatric data must be used for *Z*-score calculation, which are available for children older than 3 years for the TBLH, while LS measurements are feasible and reproducible also for children aged < 3 years [81].

Pitfalls in DXA measurement are common as it is a 2-dimensional technique. For example, DXA measurements underestimate BMD (g/cm^2^) in children with short stature or pubertal delay and appropriate methods to account for growth delay when interpreting DXA results for children < 5 years are currently unknown. Hence, adjustment for bone size or skeletal size is mandatory. To do so, volumetric BMD (vBMD or bone mineral apparent density [BMAD], g/cm^3^) is calculated or BMD *Z*-scores are adjusted for height [27, 82]. In addition, disrupting factors, such as movement during measurement, scoliosis, and metalwork, can give non-interpretable results [83]. If DXA-LS cannot be performed, alternative sites are the distal forearm, the proximal hip, and the lateral distal femur [30]. Despite these pitfalls, DXA remains the technique of choice to measure bone mass.

### Radiogrammetry

To assess bone health on digital radiographs, different parameters have been proposed such as the Bone Health Index® (BHI) [84, 85]. Studies comparing BHI determined by X-ray and BMD assessed by DXA showed contradictory results [86–92]. BHI seems to overestimate bone health impairment, also, the correlation with DXA measurements is not always good as it applies mainly to absolute values than to *Z*-scores. Therefore, its use is not currently recommended.

### Radiography

**Fig. 1:**
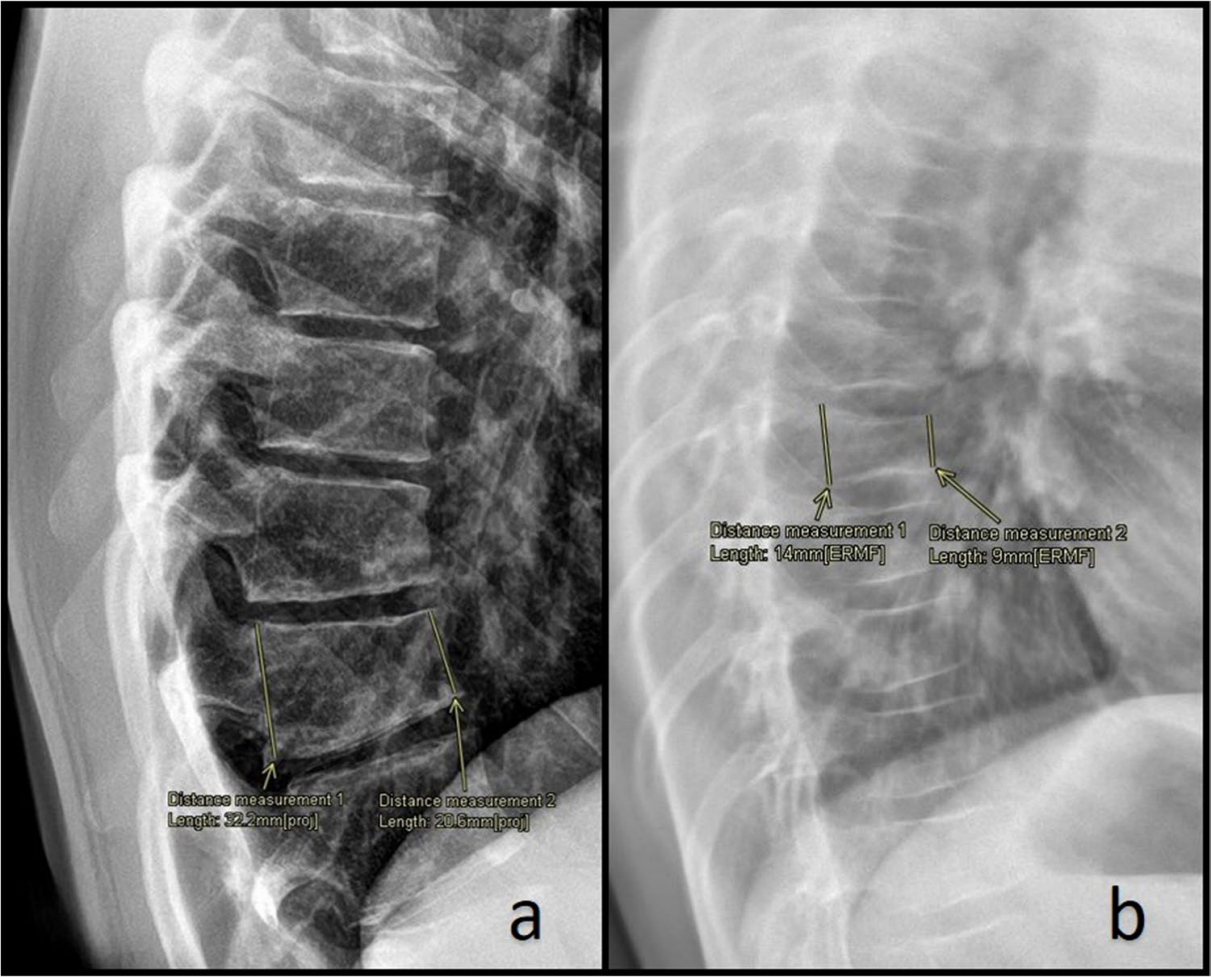
**a** Male adolescent with backpain, no history of trauma reported. Lateral radiograph of the spine shows a vertebral fracture of the 12^th^ thoracic vertebra. There is a 35% loss of height, in keeping with a grade 2 fracture according to the Genant classification (moderate fracture, 25 to 40% loss of height). The 11^th^ thoracic vertebra and the 1^st^ lumbar vertebra also show mild wedging; measurements are not shown to prevent clutter of the image. **b** Girl with juvenile osteoporosis. Lateral radiograph of the spine shows multiple fractures. Measurement shows a 34% loss of height, in keeping with a grade 2 fracture according to the Genant classification (moderate fracture, 25 to 40% loss of height)

**Fig. 2:**
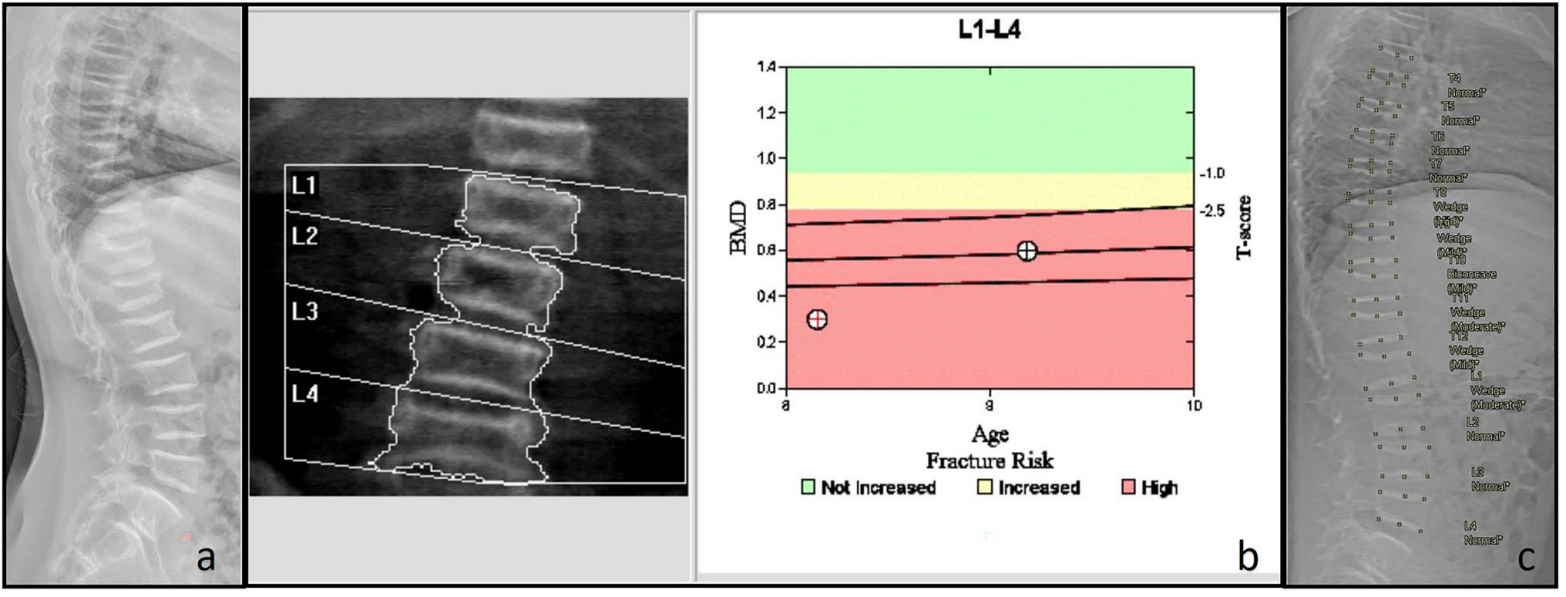
Infant with Osteoporosis-pseudoglioma syndrome (LRP5 mutation) treated with bisphosphonates. **a** Lateral spine radiograph shows multiple vertebral fractures of the thoracic and lumbar spine. There are dense vertebral endplates as a result of bisphosphonate treatment. **b** Although DXA of the lumbar spine shows a low BMD, it is underestimating the severity of the disease due to the loss of height and the increased density of the vertebral endplates. **c** Automated DXA vertebral fracture assessment (VFA)

Radiography is used to detect VFs and scoliosis. In contrast to adult guidelines, where back pain alone does not represent an indication to perform imaging, lateral spine imaging (thoracic and lumbar vertebrae) should be performed in all children with suspected osteoporosis and hidden VFs should be investigated [93, 94]. VFs are usually assessed through the Genant’s semi-quantitative method. This method is based on the estimation of the vertebral height loss and the visual evaluation of morphological change. A vertebral height loss > 20% indicates a VF, with 20–25% height loss defined as mild, 26–40% as moderate, and > 40% as severe (Fig. 1) [95–97]. Recent studies have shown that the newest generation of DXA scanners can also detect moderate to severe VFs in children through the vertebral fracture assessment (VFA), using a lower doses of radiation than lateral spine radiographs (Fig. 2) [98–100].

### Quantitative computed tomography

Quantitative computed tomography (QCT), peripheral QCT (pQCT), and vertebral QCT (vQCT) are able to assess cortical and trabecular bone separately, vBMD rather than aBMD and provide information on bone geometry, impossible to obtain with DXA [62].

pQCT devices evaluate bone at the level of radius or tibia [101] and can be used as an alternative in children with severe scoliosis that cannot undergo DXA-LS. Main limitations are related to the need of proper positioning of the patient to achieve reproducibility and movements during the scan can result in artefacts. Furthermore, whether pQCT measurements adequately reflect the whole skeleton, including the spine, is under debate. Reference data are available [102–104] but have their limitations [105].

### Other diagnostic techniques

Magnetic resonance imaging (MRI) is not routinely used in clinical practice yet, but presents several advantages. MRI provides volumetric bone measures and can separately evaluate cortical and trabecular bone like QCT. In addition to QCT, MRI can scan both axial and peripheral skeleton at the same time and acquire data from multiple anatomical planes with no need to reposition the patient. However, the time of acquisition of images is long (around 20–30 min), the closed space could cause stress, sedation is required for younger children, and the costs are high [106].

Quantitative ultrasonography (QUS) has been proposed as a useful tool to assess bone mineral status from early childhood to young-adulthood with a very small confounding effect related to bone size [107, 108]. Nevertheless, its use is not recommended in pediatric population with the exception of research settings [109, 110].

In children with a history of frequent low impact fractures and unclear causation, a trans-iliac bone biopsy with tetracycline double-labeling may be indicated. Structural and dynamic parameters of bone quality can be obtained through histomorphometry and tissue density assessed using backscattered electron microscopy. Bone biopsy, however, is infrequently performed because it is invasive, requires anesthesia, and is performed only in specialized centers [7, 49, 111].

## Conclusion

Increased awareness among pediatricians is important as both genetic and acquired pediatric bone disorders cause substantial morbidity and require early detection. Osteoporosis in children requires consultation with and/or referral to a pediatric bone specialist. This is particularly relevant since children possess the unique ability for spontaneous and medication-assisted recovery, including reshaping of vertebral fractures. As such, pediatricians have an opportunity to improve bone mass accrual and musculoskeletal health in osteoporotic children.

**Fig. 3:**
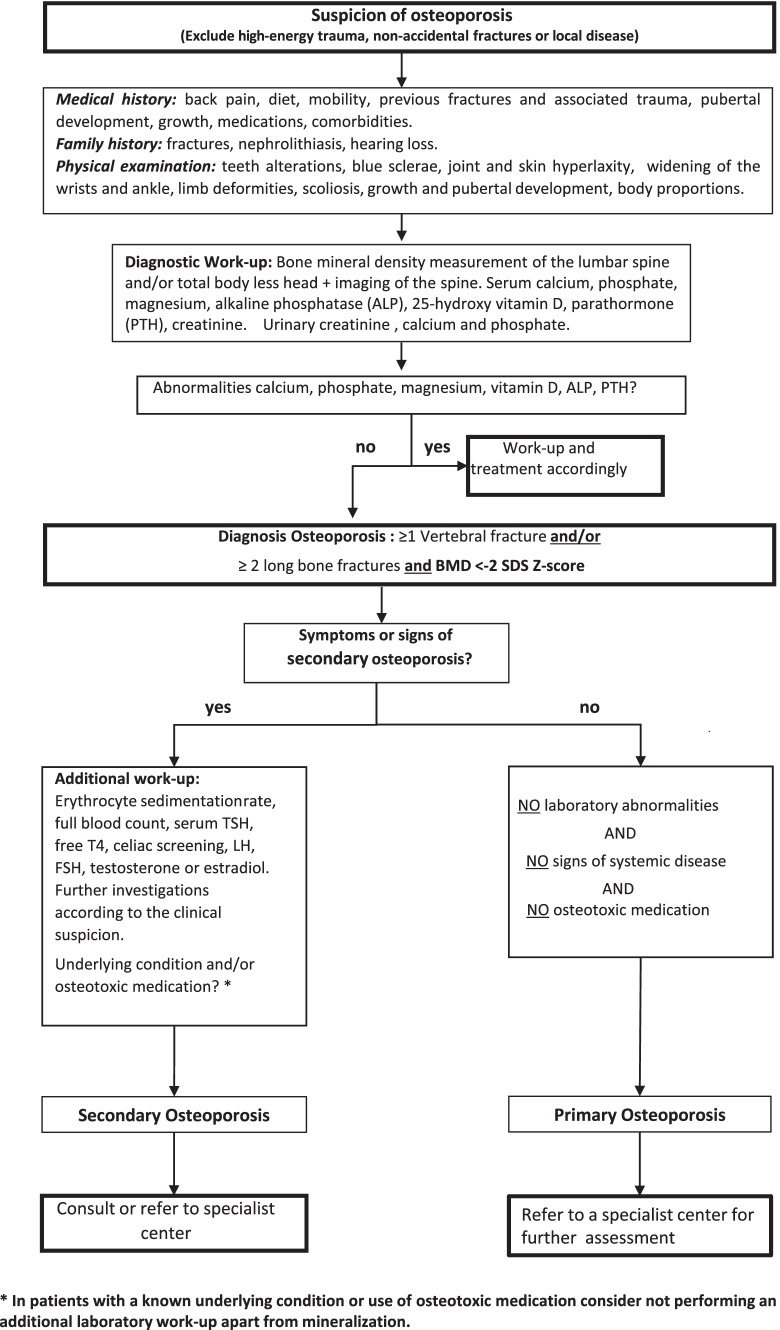
Diagnostic work-up in a child suspected of osteoporosis

OI represents the main cause of primary osteoporosis, but many more rare genetic conditions affecting bone health are recognized that require specialist management. In children at risk of primary and secondary osteoporosis, it is mandatory to check for vertebral fractures. At first presentation, a careful history and examination should be taken and bone hypomineralization disorders excluded by laboratory investigations. To date, DXA and conventional X-radiographs are the techniques of choice to assess bone health and diagnose vertebral fractures. We emphasize that DXA interpretation requires pediatric expertise and discourage DXA use in children outside specialist centers. The flowchart in Fig. 3 summarizes the main steps that the pediatricians should take when evaluating a child suspected of bone fragility.

## Data Availability

All articles included in the review are cited among the references.

## Abbreviations

ALP: Alkaline phosphatase
BHI: Bone health index
BMAD: Bone mineral apparent density
BMD: Bone mineral density
DXA: Dual-energy x-ray absorptiometry
VFs: Vertebral fractures
GGT: Gamma glutamyl transferase
GCs: Glucocorticosteroids
ICS: Inhaled corticosteroid
IJO: Idiopathic juvenile osteoporosis
IL: Interleukine
LRP: Low-density lipoprotein receptor-related protein
LS: Lumbar spine
MRI: Magnetic resonance imaging
OI: Osteogenesis imperfect
OPG: Osteoprotegerin
pQCT: Peripheral QCT
PTH: Parathyroid hormone
QCT: Quantitative computed tomography
QUS: Quantitative ultrasonography
RANK: Receptor activator of nuclear factor kappa B
RANKL: RANK ligand
TBLH: Total (whole) body less head
TGF-β: Transforming growth factor-β
vBMD: Volumetric BMD
vQCT: Vertebral QCT
WNT: Wingless iNTegration site family
